# Disruption of STAT5A and NMI signaling axis leads to ISG20-driven metastatic mammary tumors

**DOI:** 10.1038/s41389-021-00333-y

**Published:** 2021-06-02

**Authors:** Heba Allah M. Alsheikh, Brandon J. Metge, Hawley C. Pruitt, Sarah C. Kammerud, Dongquan Chen, Shi Wei, Lalita A. Shevde, Rajeev S. Samant

**Affiliations:** 1 Department of Pathology, University of Alabama at Birmingham, Birmingham, AL USA; 2 Department of Medicine, University of Alabama at Birmingham, Birmingham, AL USA; 3 O’Neal Comprehensive Cancer Center, University of Alabama at Birmingham, Birmingham, AL USA; 4 Birmingham VA Medical Center, Birmingham, AL USA

**Keywords:** Breast cancer, Metastasis

## Abstract

Molecular dynamics of developmental processes are repurposed by cancer cells to support cancer initiation and progression. Disruption of the delicate balance between cellular differentiation and plasticity during mammary development leads to breast cancer initiation and metastatic progression. STAT5A is essential for differentiation of secretory mammary alveolar epithelium. Active STAT5A characterizes breast cancer patients for favorable prognosis. N-Myc and STAT Interactor protein (NMI) was initially discovered as a protein that interacts with various STATs; however, the relevance of these interactions to normal mammary development and cancer was not known. We observe that NMI protein is expressed in the mammary ductal epithelium at the onset of puberty and is induced in pregnancy. NMI protein is decreased in 70% of patient specimens with metastatic breast cancer compared to primary tumors. Here we present our finding that NMI and STAT5A cooperatively mediate normal mammary development. Loss of NMI in vivo caused a decrease in STAT5A activity in normal mammary epithelial as well as breast cancer cells. Analysis of STAT5A mammary specific controlled genetic program in the context of NMI knockout revealed ISG20 (interferon stimulated exonuclease gene 20, a protein involved in rRNA biogenesis) as an unfailing negatively regulated target. Role of ISG20 has never been described in metastatic process of mammary tumors. We observed that overexpression of ISG20 is increased in metastases compared to matched primary breast tumor tissues. Our observations reveal that NMI-STAT5A mediated signaling keeps a check on ISG20 expression via miR-17–92 cluster. We show that uncontrolled ISG20 expression drives tumor progression and metastasis.

## Introduction

Distorted manifestation of developmental processes, such as cellular plasticity, are used by cancer cells to support cancer initiation and progression to metastatic disease^1,2^. Luminal and basal are two types of epithelia in the mammary ducts. The luminal epithelium forms the inner lining of the ducts and the secretory alveoli, these cells produce mik. Whereas the basal epithelium consists of myoepithelial cells that help secretion of milk out of the alveolar sacks^3,4^. During pregnancy, luminal progenitor cells epithelial cells promptly divide and undergo detailed changes in terminal end bud area to form mature alveoli that secrete milk at parturition^5^. In normal mammary luminal epithelium, proliferation of differentiated ERα-positive epithelial cells and their stimulation of ERα-negative cells is strictly regulated, but in breast cancer this control is lost^6,7^. Thus, it is likely that there are distinct factors that maintain homeostasis of differentiated alveolar cells during pregnancy, but fail to act during tumor mammary progression^4^.

Our recent findings show that N-Myc and STAT interactor (NMI) is expressed in the mammary ductal epithelium at the onset of puberty and is induced in pregnancy and lactation. NMI expression is critical for ensuring the maintenance of differentiated luminal epithelial cells^8^. Our mammary-specific NMI knockout mouse revealed that NMI loss disrupts luminal differentiation in the mammary glands affecting alveologenesis and prompts the progression of tumors with aggressive metastatic characteristics^8^. In agreement with this, NMI protein expression is significantly decreased in 70% of patient specimens with metastatic breast cancer compared to primary tumors^8,9^.

Members of the signal transducer and activator of transcription (STAT) protein family are intracellular transcription factors that mediate many aspects of cellular differentiation, proliferation, apoptosis, and immunity. The STAT family of proteins is implicated in mammary gland development and is also commonly involved in breast tumorigenesis^10^. NMI interacts with all STATs except STAT2^11^. STAT5 specifically has a fundamental role in development of mammary epithelial cells during pregnancy^12^, and its loss results in lactation failure in pregnant mice^13^.

Here we present our finding that NMI and STAT5A have a cooperative relationship in normal mammary development. We demonstrate that silencing NMI decreases STAT5A activity in undifferentiated murine mammary epithelial cells and leads to a subsequent failure to differentiate. Detailed examination of STAT5A controlled mammary-specific transcription program in comparison with transcription profiles of NMI expressing or silenced models revealed ISG20, interferon stimulated exonuclease gene 20, as a unique negatively regulated transcript. Here we show that ISG20 expression is increased in metastatic tissues compared to their matched primary breast cancer tissues. We elucidate that NMI and STAT5A keep ISG20 in check by through microRNA hsa-miR-20a. We also show that ISG20 has a positive influence on tumor progression and metastasis.

## Results

### NMI and STAT5A show concurrent expression pattern during mammary gland differentiation

**Fig. 1:**
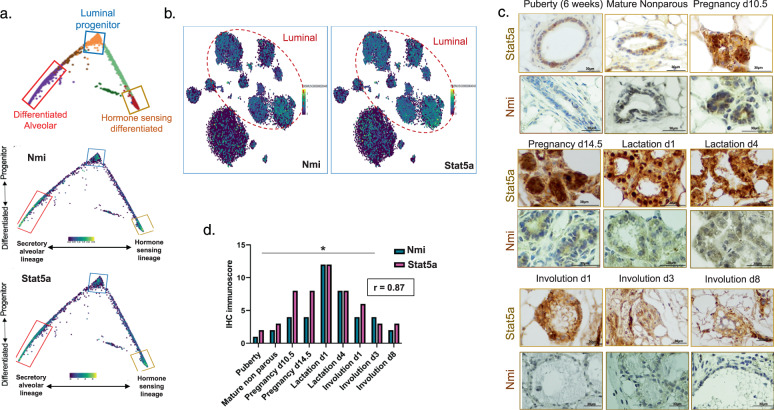
NMI and STAT5A show concurrent expression pattern during mammary gland differentiation. **a** NMI and STAT5A expression within computational reconstruction of the luminal compartment generated from single-cell RNA-seq mouse mammary cells different stages of differentiation. **b** t-distributed stochastic neighbor embedding (t-SNE) overlaid NMI and STAT5A expression in different Luminal and Basal clusters. **c** Immunohistochemical staining for STAT5A and NMI on mouse mammary tissues during different stages of development (puberty, mature non parous, pregnancy d10.5, pregnancy d14.5, lactation d1, lactation d4, involution d1, involution d3, and involution d8). **d** IHC immunoscore for STAT5A and NMI staining on mouse mammary tissues during differentiation. Expression was the highest during pregnancy and lactation, then subsided during the course of involution. The pattern of expression showed a significant positive correlation between NMI and STAT5A.

NMI has a distinct role in the differentiation of luminal progenitor cells^8^. Similarly development of mammary luminal progenitor cells is controlled by the transcription factor STAT5A^14^. We examined the expression of NMI and STAT5A in single-cell RNA sequencing of the luminal differentiation trajectory of mouse mammary epithelial cells (MECs)^15^. Transcripts for both genes showed enrichment in three differentiated clusters; the luminal progenitor, differentiated alveolar, and hormone sensing (Fig. 1a). Additionally, we observed their enriched expression in the mouse mammary gestation cluster of luminal cells (Fig. 1b). This observation pointed to concurrent expression patterns of these genes in mammary tissue. To see if protein expression patterns are also similar, we stained mouse mammary tissues at different stages of development. NMI levels peak in mammary tissues during pregnancy and lactation^8^. Expression of STAT5A protein reaches its peak during pregnancy and is sustained during lactation. STAT5A levels start to decline during involution (Fig. 1c). This pattern of expression showed a strong positive correlation with the expression of NMI in the same tissue samples. (r = 0.87). (Fig. 1d**)**. We decided to further investigate if there is a functional relationship behind these correlative observations.

### NMI expression coincides with active STAT5A in HC11 mammary cell differentiation

**Fig. 2:**
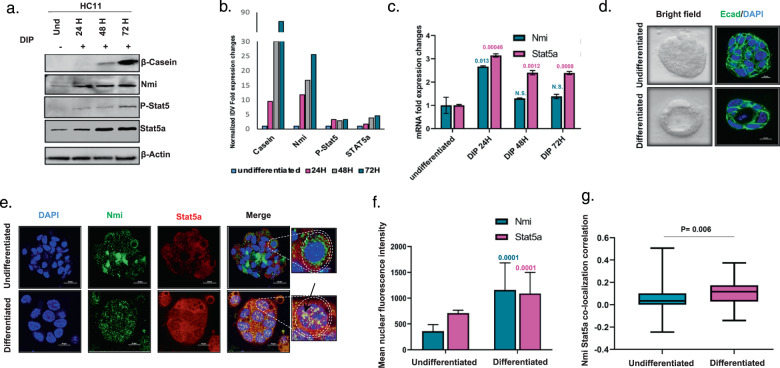
NMI and STAT5A show concurrent expression pattern and nuclear localization in HC11 mammary cell differentiation. **a** Western blot for β-Casein, NMI, Phospho-STAT5A, and STAT5A in undifferentiated HC11 cells and during the induction of differentiation using Dexamethasone, Insulin and Prolactin at hours 24, 48, and 72 h from the start of induction. **b** Normalized integrated density values for western blot signals for β-casein, NMI, P-STAT5A, and STAT5A in HC11 during the course of differentiation. **c** Fold expression changes of NMI and STAT5A using RTq-PCR in undifferentiated HC11 cells and during the induction of differentiation using Dexamethasone, Insulin, and Prolactin at hours 24, 48, and 72 from the start of induction. **d** Undifferentiated HC11 cells that were embedded in Matrigel and stimulated by DIP to differentiate into organized mammary acini. Right panel represents bright field images and left panel represents fluorescence imaging of E-cadherin in green to represent cell-to-cell junction and DAPI staining for nucleus in blue. **e** Immunofluorescence staining for NMI (Green) and STAT5A (red) in undifferentiated and differentiated HC11. DAPI was used to stain the nucleus, magnified pictures represent nuclear translocation of NMI and STAT5A in differentiated cells. **f** Graph represents mean nuclear florescence intensity quantification of NMI and STAT5A in undifferentiated and differentiated cells. This quantitation was conducted for all cells in the Z-stack of 3D organoid. **g** Graph represents Nmi and STAT5A colocalization correlation in nuclei of undifferentiated (*n* = 20) and differentiated (*n* = 15) cells.

Undifferentiated mouse mammary epithelial cells (HC11) are a convenient model to study key molecular aspects of development of mammary epithelial cells. Differentiated HC11 cells produce β-casein, which is the main protein component of milk secreted by mammary acini. We noticed that during the process of HC11 differentiation, the expression of STAT5A, its active form P-STAT5 together with NMI were elevated concurrently with differentiation stimulus (Fig. 2a, b). RNA levels of STAT5A were also elevated as early as 24 h post-differentiation induction (Fig. 2c). To further investigate the dynamics of STAT5A and NMI expression during differentiation, HC11 cells were cultured in 3D and induced to differentiate into mammary acini (Fig. 2d). Nuclear translocation of STAT5 is a key to its function and indicates its activity^16^. We noticed that differentiated mammary acini showed simultaneous upregulation and colocalization of STAT5A and NMI in the nucleus of the mammary cells (Fig. 2e). This nuclear enrichment of the two molecules is significant as seen in Fig. 2f. Moreover, Nmi and Stat5a signals colocalize within the nucleus (Fig. 2g). Overall, our findings revealed concurrent increase in NMI and active STAT5A during mammary differentiation and prompted us to investigate if there is a common signaling network governed by these proteins.

### Silencing NMI impedes JAK/STAT signaling mediated mammary differentiation

**Fig. 3:**
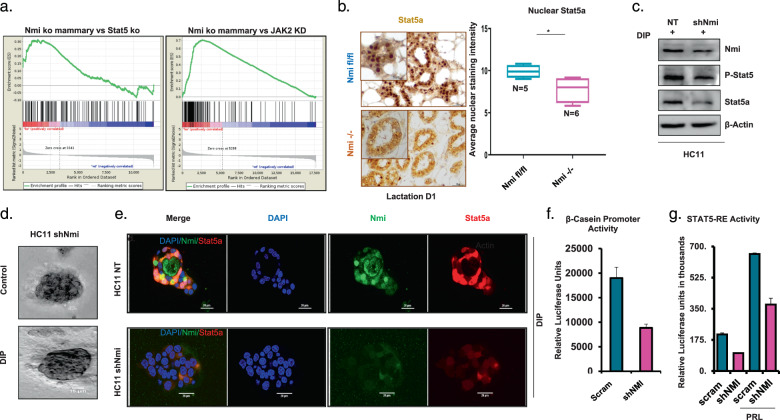
Silencing NMI hindered JAK/STAT signaling mediated mammary differentiation. **a** GSEA of NMI wild-type compared to NMI knockout mammary mouse tissues at day 1 of lactation in comparison to list of genes upregulated after knockdown of JAK2 in HEL cells (left panel) and in comparison, to genes affected in lactating mouse mammary tissues after deletion of STAT5 enhancers (Right panel). **b** Immunohistochemical staining for STAT5A on mouse mammary tissues at lactation d1 in NMI fl/fl (*n* = 5) vs NMI −/− (*n* = 6) mice. Graph represents quantified nuclear STAT5A staining intensity, which is significantly lower in NMI−/− mice tissues (*p* = 0.017). **c** Western blot of Nmi, P-STAT5A, STAT5A in nontransfected control vs shNMI HC11 cells. **d** Undifferentiated shNMI HC11 cells that were embedded in Matrigel and stimulated by DIP to differentiate shNMI cells failed to differentiate into organized mammary acini. **e** Immunofluorescent staining for NMI (Green) and STAT5A (red) in control and shNMI HC11 cells that were embedded in Matrigel and stimulated to differentiate with DIP. DAPI was used to stain the nucleus. Pictures show loss of NMI and STAT5A nuclear translocation after knocking down NMI. **f** Luciferase assay for measuring activity of β-casein promotor. Data show decreased activity in T47D shNMI cells (*p* = 0.08). **g** STAT5 response element (STAT5-RE) activity was measured by a STAT5 response element reporter luciferase assay with and without prolactin. Data show diminished activity in T47D shNMI cells after prolactin stimulation.

We analyzed gene expression profiles of NMI knockout mice mammary tissues using GSEA. We observed that gene set corresponding to expression profile of lactating mammary glands of STAT5 enhancer deleted mice was enriched^17,18^. Furthermore, we found a comparable pattern of enrichment with a geneset of JAK2 knocked down erytholeukemia cells^19,20^ (Fig. 3a). JAK2 phosphorylates STAT5 and thus is an activator of STAT5 signaling^21^. These observations point that mammary cells lacking NMI protein show high level of concurrence with suppressed states of JAK2 and STAT5 signaling. This indicating that there is a level of commonality in the impact of lack of NMI or inactive STAT5/JAK2 signaling.

To further solidify the NMI-STAT5 signaling relationship, we queried mammary tissues from mammary-specific NMI knockout mice for STAT5A activity. We detected that there was an overall lower expression of STAT5A and the protein was diffused throughout the cell compared to the control tissue. The nuclear localization STAT5A corresponds to its transcriptionally active state. We noticed that STAT5A nuclear localization was significantly diminished in mammary acini of NMI knockout mice (*n* = 6) compared to wild-type mice (*n* = 5) (*p* = 0.017) (Fig. 3b). This lack of nuclear localization is consistent with the reduced STAT5A transcription activity in absence of NMI per our GSEA. Moving forward, we silenced HC11 cells for expression of NMI. We noticed that compared to the control, the NMI-silenced cells show much reduced P-STAT5A levels (Fig. 3c). Additionally, in 3D Matrigel culture, NMI-silenced HC11 cells failed to form organized mammary acini (Fig. 3d). P-STAT5A is transcriptionally active form of STAT5A and is localized to the nucleus. We noticed that in the 3D model of HC11, silencing NMI not only lowered overall STAT5A levels but also reduced STAT5A level in the nucleus in response to differentiation stimulation (Fig. 3e) (Supplementary Fig. 1). These observations endorse the findings we established from NMI knockout mouse mammary tissues reaffirming that mammary cells lacking NMI protein show impediment of STAT5A signaling activity.

We utilized luciferase plasmids bearing either proximal promoter (β-Casein) or tandem repeat GAS sequences (STAT5 response element) to test if absence of NMI can impact STAT5 transcription. We observed that T47D breast cancer cells stably silenced for NMI expression showed significantly reduced activities of β-Casein promoter and STAT5 response element reporter (Fig. 3f, g). Overall, our observations reveal that NMI and STAT5A have a unique cooperative relationship in mammary development. This prompted us to investigate if loss of NMI expression in metastatic breast cancer influenced STAT5A status and activity.

### NMI/STAT5A axis is downregulated in breast cancer and its expression is indicative of reduced metastasis and better prognosis

**Fig. 4:**
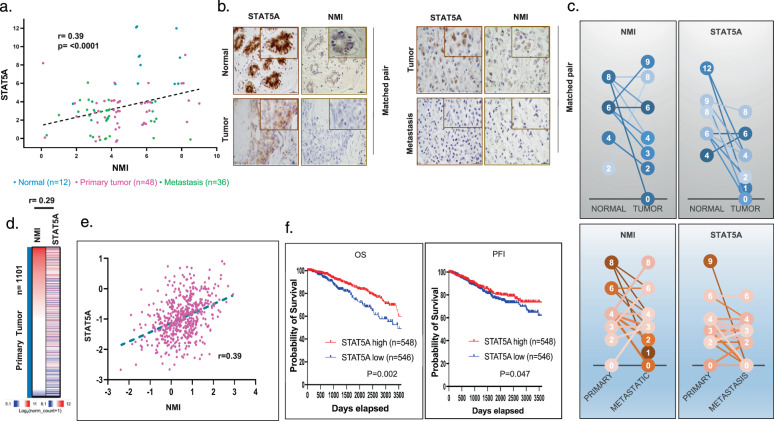
NMI/STAT5A axis is downregulated in breast cancer and its expression is distinctive for less frequent metastasis and good prognosis. **a** Correlation of STAT5A and NMI immunoscore in human breast cancer TMA of normal breast tissues (*n* = 12), primary breast tumors (*n* = 48), and metastatic LN (*n* = 36), graph represents a significant moderate correlation between NMI and STAT5A score (r = 0.39) (*p* ≤ 0.0001). **b** Immunohistochemical staining for STAT5A and NMI on matched normal breast vs primary breast tumor (left panel) and matched primary breast tumor vs metastatic LN (right panel) **c** Immunoscore for STAT5A and NMI from matched human normal breast vs primary breast tumor and matched primary breast tumor vs metastatic LN. (Normal vs tumor NMI (*P* = 0.17) STAT5A (*p* = 0.001)) (Primary vs metastatic NMI (*p* = 0.05) STAT5A (*p* = 0.004)). The number in the circle represents immunoreactive score. The shade of color for individual circle is assigned it to distinguish matched sample. The line connects the matched samples from the same patients. **d** Heatmap of NMI, STAT5A, and JAK2 RNA-seq expression in TCGA 1101 primary human breast cancer. **e** Graph represents significant positive correlation between expression of STAT5A and NMI in TCGA (r = 0.39) (*p* ≤ 0.0001). **f** TCGA data-based KM survival curve for STAT5A RNA-seq low and high-expressing patients. Graph shows significantly higher overall survival (OS) (*p* = 0.002) and Progression-free interval (PFI) (*p* = 0.047) in STAT5A high-expressing patients.

In order to investigate the relationship of NMI and STAT5A protein expression with breast cancer progression, we immunostained normal breast tissues and breast cancer tissues for NMI and STAT5A. We noticed a significant positive correlation between NMI and STAT5A protein expression (r = 0.39, *p* ≤ 0.0001) (Fig. 4a). Normal (uninvolved) breast tissues showed elevated protein expression for NMI and STAT5A compared to their matched primary tumor tissues (Fig. 4b, c). More interesting is that primary tumors showed increased expressions of NMI and STAT5A compared to their corresponding lymph node (LN) metastasis (Fig. 4b, c). Consistent with these observations, analysis of mRNA expression data from 1101 primary breast tumors showed a weak positive correlation between NMI and STAT5A expression (Fig. 4d, e). We analyzed publicly available (TCGA) RNA-seq datasets and found that patients with high STAT5A mRNA expressing breast tumors had a significantly higher overall survival (*p* = 0.002) and progression-free interval (*p* = 0.047) compared to patients with tumors that have low STAT5A (Fig. 4f). Overall, our observations from breast cancer patients compelled us to further investigate the consequences of diminished STAT5A and NMI expression during the process of tumorigenesis and metastasis.

### NMI/STAT5A axis downregulates ISG20 through miRNA hsa-miR-20

**Fig. 5:**
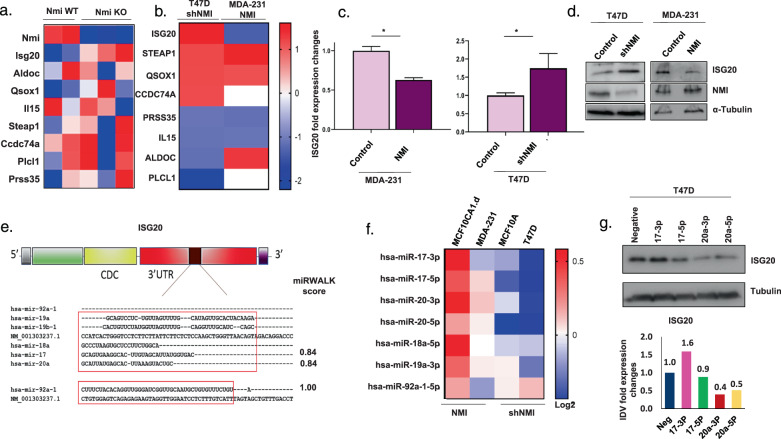
NMI/STAT5A axis downregulate ISG20 through miRNA hsa-miR-20. **a** Heatmap of RNA-seq expression of STAT5 enhancer related genes in mammary mouse tissues for wild-type (*n* = 2) vs NMI KO (*n* = 3) mice mammary tissue. **b** Heatmap of fold change RNA-seq expression of Stat5 enhancer related genes in T47D shNMI vs control and 231 NMI vs control. **c** Fold expression changes of ISG20 using RTq-PCR in T47D vector control and shNMI, MDA-MB-231 scrambled control and NMI, ISG20 expression was significantly higher in T47D shNMI (*p* = 0.03) and lower in MDA-MB-231 NMI (*p* ≤ 0.0001). **d** WB of ISG20 and NMI proteins in T47D control and shNMI and MDA-MB-231 control and NMI expressing cells. **e** Schematic illustration of ISG20 gene structure with predicted binding sites of miR-17–92 cluster at the 3’UTR region. **f** Heatmap representing fold changes of miR-17–92 cluster expression in miRNA array for MCF10CA1cl.D NMI, MDA-MB-231 NMI, MCF10A shNMI, and T47D shNMI compared to controls, scale represents log2 fold changes. **g** Western blot of ISG20 in T47D cells 48 h after being supplemented with miRNA mimics (100 nM) Hsa-miR-17-3p, Hsa-miR-17-5p, Hsa-miR-20a-3p, or Hsa-miR-20a-5p. Graph represents IDV quantification fold changes.

Using GSEA, we observed that geneset corresponding to expression profile of lactating mammary glands of STAT5 enhancer deleted mice (reported by Hennighausen group^17,18^) was enriched in comparison with gene expression profiles NMI knockout mice mammary tissues (Fig. 3a). This indicated that there may be a common set of genes regulated by STAT5A and NMI. Hence, we examined the enriched gene signature from the GSEA with RNA-seq data obtained from NMI wild-type and knockout mammary tissue (Fig. 5a)**;** as well as with RNA-seq data obtained from overexpression and silencing of NMI in human breast cancer cell lines MDA-MB-231 and T47D, respectively, to determine if some consistent common genes emerge (Fig. 5b). Interferon Stimulated Exonuclease Gene 20 (ISG20) consistently showed an inverse expression profile with respect to NMI. This pattern of expression was confirmed using specific RTq-PCR for ISG20 (Fig. 5c). ISG20 protein levels increased in T47D cells silenced for NMI and decreased in MDA-MB-231 cells ectopically overexpressing NMI (Fig. 5d). All these observations provide a strong indication that NMI might be negatively regulating ISG20 in breast cancer cells. We wanted to understand the mechanistic details of this regulation.

We used a web-based prediction algorithm to identify miRNAs that are predicted to target ISG20^22^. We identified that members of the miR-17–92 cluster have predicted binding sites in ISG20 (Fig. 5e). This cluster maps to human chromosome 13 and encodes six individual miRNAs (miR-17, miR-18a, miR-19a, miR-20a, miR-19b-1, and miR-92a)^23^. Of note, this miR-17–92 cluster is regulated by STAT5^24^. Using siRNA, we silenced STAT5A in MDA-MB-468 breast cancer cells. This caused an overall reduction in all the members of the miR-17–92 cluster (Supplementary Fig. 2a, b) with a concomitant increase in ISG20 RNA as well as protein expression (Supplementary Fig. 2c, d). We subjected NMI-silenced T47D and MCF10A cells and MCF10CA1.d and MDA-MB-231 cells ectopically overexpressing NMI, to global microRNA expression profiling using miRCURY LNA™ microRNA Array. We observed that members of miR-17–92 cluster were downregulated in NMI-silenced cells whereas they were upregulated in NMI overexpressed. (Fig. 5f).

We overexpressed miR-17–92 cluster in MDA-MB-468 cells (Supplementary Fig. 2e) and noticed a reduction in ISG20 protein expression (Supplementary Fig. 2f). To further investigate which member of this miRNA cluster family targets and negatively regulates ISG20, we independently introduced miRNA mimics (chemically modified ds-RNAs that mimic endogenous miRNAs and enable miRNA functional analysis by upregulation of miRNA activity) of hsa-miR-17-3p, hsa-miR-17-5p, hsa-miR-20-3p, and hsa-miR-20-5p in T47D cells. These members were specifically chosen due to their high miRWALK score (Fig. 5e). Both, hsa-miR-20-3p and hsa-miR-20-5p markedly decreased ISG20 protein level, while hsa-miR-17-3p and has-miR-17-5p did not have a significant impact on ISG20 level in T47D cells (Fig. 5g). This indicates that hsa-miR-20-3p and −5p execute an effective check on ISG20 expression.

### ISG20 expression negatively correlates with STAT5A, and its elevated expression promotes aggressive metastatic phenotype

**Fig. 6:**
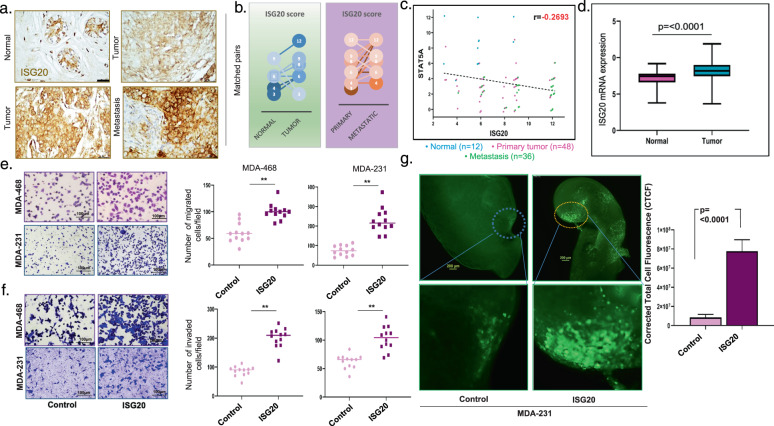
ISG20 negatively correlates with STAT5A and its expression promotes aggressive metastatic phenotype. **a** Photomicrographs showing immunohistochemical staining for ISG20 using matched normal breast compared with corresponding primary breast tumor (left panel) and matched primary breast tumor compared with corresponding metastatic LN (right panel). **b** Immunoscore of ISG20 staining comparing matched human normal breast vs primary breast tumors and matched primary breast tumors vs corresponding metastatic LN. (Average IRS score 6.0 in normal vs 7.2 in tumor (*p* = 0.15)) (7.7 in Primary vs 9.5 in metastatic (*p* = 0.0002)). **c** Correlation between STAT5A and ISG20 immunoscores in human breast cancer TMA consisting of normal breast tissues (*n* = 12) primary breast tumors (*n* = 48) and metastatic LN (*n* = 36), graph represents a significant negative correlation between ISG20 and STAT5A score (r = 0.27) (*p* = 0.008). **d** ISG20 mRNA expression from TCGA data (RNA-seq) for breast cancer- normal breast tissues and breast tumor tissues are compared. ISG20 expression is significantly higher in breast cancer compared to normal breast tissue (*p* ≤ 0.0001). **e** Representative images of transmembrane migration for control or MDA-MB-468 and MDA-MB-231 cells overexpressing ISG20. Graphs represent significantly higher migrated cells/field in ISG20 expressing MDA-MB-468 (*p* = 0.0003) and MDA-MB-231 (*p* ≤ 0.0001). **f** Representative images for control or ISG20 MDA-MB-468 and MDA-MB-231 cells Invading through Matrigel invasion chamber. Graphs represent significantly higher invaded cells/field in ISG20 MDA-MB-468 (*p* ≤ 0.0001) and MDA-MB-231 (*p* ≤ 0.0001). **g** Images from fluorescence labelled MDA-MB-231 control or ISG20 overexpressing cells in PUMA assay. Nude mice were injected in the lateral tail (1 × 10^5^). Agar perfused lung slices were cultured on Surgifoam for 32 days. Metastatic burden was quantified as corrected total cell fluorescence (CTCF). MDA-MB-231 cells overexpressing ISG20 show a higher metastatic burden than the control cells (*p* ≤ 0.0001).

ISG20 is a 3′ to 5′ exonuclease for single stranded RNA including rRNA and to some extent for single stranded DNA. It plays a role in estrogen mediated cellular proliferation and differentiation^25^; however, little is known about its role in breast cancer. Thus, to investigate its role in breast cancer, we stained for ISG20 in 96 human breast samples on an array consisting of normal tissues, primary breast tumor or lymph node (LN) metastasis (Fig. 6a). ISG20 staining is mainly located at the luminal side of the alveolar region of the normal human mammary duct (Supplementary Fig. 3a). We noticed that ISG20 levels were much elevated in primary tumors in comparison to their matched normal tissues, and furthermore ISG20 expression was much higher in LN metastases versus their matched primary breast tumor (Fig. 6b). Moreover, we found a weak negative correlation (r = −0.27) between the pattern of expression of ISG20 with STAT5A within the same patient’s tissue samples (Fig. 6c). Additionally, we examined ISG20 expressions across publicly available RNA sequencing data of 1247 breast cancer patients and found that ISG20 levels are significantly higher in breast tumor tissues compared to normal mammary tissues (*p* ≤ 0.0001) (Fig. 6d).

To better understand the functional impact of elevated ISG20 expression, we overexpressed ISG20 in MDA-MB-231 and MDA-MB-468 cells using lentivirus transduction (Supplementary Fig. 3b). We observed that ISG20 overexpressing cells showed a very effective migration capability compared to the control group (*p* ≤ 0.0003) (Fig. 6e). These cells also showed remarkably higher invasive ability through Matrigel than their control counterpart (*p* ≤ 0.0001) (Fig. 6f). MDA-MB-231 IS20 overexpressing cells grown in 3D showed much reduced circularity than their controls. The ISG20 overexpressing structures show highly irregular-stellate-like pattern (Supplementary Fig. 3c). Analysis of these 3D structures showed that MDA-MB-231-ISG20 expressors made structures with significantly less circularity (Supplementary Fig. 3d). Such stellate-like (or spiky) 3D growth features indicate highly invasive phenotype^26,27^. To test this effect of ISG20 on invasive ability of cells, we knocked down ISG20 expression from MDA-MB-231 cells using shRNA (Supplementary Fig. 3e). The resulting cells displayed remarkably reduced invasive ability (Supplementary Fig. 3f, g).

Ex-vivo pulmonary metastasis assay (PuMA) is an effective tool to visually investigate metastatic lung colonization ex-vivo^28^. In this assay, following injection of fluorescently labeled cancer cells in to the lateral tail vein of mice, lungs are cannulated and perfused with agarose. Thin sections of these lungs can then be grown on a Surgifoam in tissue culture media. This allows ex-vivo monitoring of the growth of cells that lodged and colonized to lungs. We used PuMA to investigate if breast cancer cells with elevated ISG20 expression will show efficient lung colonization. Cells overexpressing ISG20 showed much higher metastatic growth compared to the control lung slices (*p* ≤ 0.0001) (Fig. 6g). Taken together our findings demonstrate metastasis promoting role of ISG20 in breast cancer.

## Discussion

The mammary gland is a composite secretory organ that contains a number of different cell types.

Luminal and myoepithelial cells form the ductal structure of the glands. During pregnancy, the mammary gland undergoes development and morphological change to prepare for lactation. Lactation requires specialized cells that can synthesize and secrete copious amounts of milk. Progesterone hormone together with prolactin promotes the differentiation of the alveoli, which are the structures that produce, store, and secrete milk during lactation^29,30^. The luminal cells line the lumen of alveoli and differentiate from luminal progenitor cells. The luminal progenitor cells are thought to give rise to all molecular subtypes of breast cancer except claudin low tumors^3^. Disrupted pathways and processes observed during breast cancer progression in many ways mimic those witnessed during normal mammary gland development and tissue remodeling^5,31^. The reasons behind the erroneous manifestation of these developmental events that promote breast cancer progression remain to be uncovered.

NMI protein has signaling influence on the mammary ductal epithelium^8^. Mammary-specific NMI knockout revealed that NMI loss disrupts luminal differentiation in the mammary gland leading to precocious alveologenesis and prompted the progression of tumors with aggressive metastatic characteristics^8^. Moreover, NMI loss promoted EMT activation in breast cancer^9^. As such, NMI loss supports tumor progression in multiple tumor types, such as breast cancer, lung adenocarcinoma, and cervical cancer^32–35^. Like many other proteins, NMI has cell and tissue-specific roles. For example, it functions as a DAMP (damage associated molecular patterns) protein and activates macrophages to release proinflammatory cytokines through the Toll-like receptor 4 pathway^36^. NMI is contextually activated by interferon and interleukin signaling and thus its close association with STAT signaling is intuitive^11,35,37^. However, the cell and tissue-specific molecular networks allow NMI-STAT signaling to regulate diverse cellular fates, including cancer stem cell traits^33^. Thus, to unravel the intricacies of STAT signaling involvement in mammary development and breast cancer our studies focused on mammary development specific events. STAT5A is one of the downstream effectors of prolactin and is essential for differentiation of secretory alveolar epithelium. In normal mammary tissue, STAT5A is much more abundant than STAT5B and constitutes more than 70% of STAT5 levels^38^. While STAT5A is a major driver of mammary development, several key questions relevant to breast cancer, remain to be answered. Phosphorylated STAT5A (active) fosters cellular differentiation and hinders invasive features of human breast cancer cell lines^39–41^. Active STAT5A characterizes breast cancer patients with favorable prognosis^42^. Findings from the study conducted by Dr Rui’s group support the notion that STAT5A signaling is frequently absent during breast cancer progression and loss of nuclear phospho-Stat5 is associated with poor prognosis in node-negative breast cancer^43,44^.

We describe a concurrent expression trend of STAT5A and NMI during sequential stages of mammary development that is reflected in molecular pictures that are evident from single-cell profiles of these stages^15^. These stages are thought to be mainly triggered by stimuli such as prolactin, responsible for remodeling the mammary gland to prepare for lactation. We show that NMI and STAT5A translocate to the nucleus upon stimulation by prolactin. STAT5A translocation to the nucleus is indicative of its active state. It was interesting to notice NMI translocation to the nucleus upon stimulation by prolactin. This relocation of NMI has not been described before. JAK2, a receptor for prolactin, self-activates and phosphorylates STAT5A on positionally conserved tyrosine residues. In turn, active STAT5A translocates to the nucleus to specific regulatory DNA elements^21^. Geneset enrichment analysis of mammary tissues from NMI-KO mice showed loss of enrichment of JAK2 pathway, which is upstream of STAT5 signaling. Nuclear STAT5A levels are diminished in mammary tissues from NMI-KO mice. This indicates reduction of STAT5A activity, which is mandatory to maintain mammary differentiation. Thus, it is logical that undifferentiated mammary cells that are silenced for NMI failed to form organized mammary acini structures in 3D culture. Taken together we demonstrate a vital functional role of NMI/STAT5A axis in differentiation of mammary epithelial cells.

**Fig. 7:**
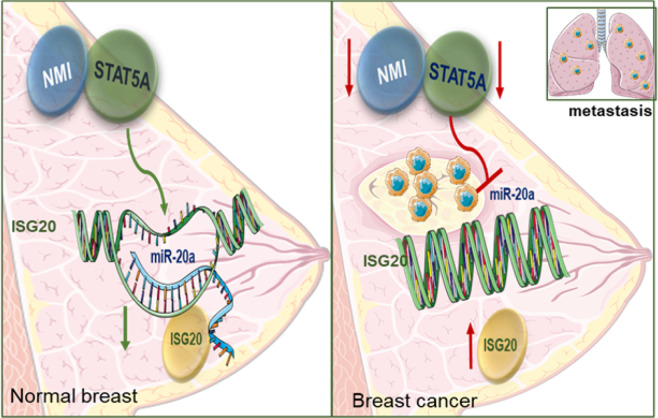
Schematic summary of findings. In normal breast NMI and STAT5A signaling keeps ISG20 expression in check, through expression of miR-20a and cells undergo normal differentiation. In cancer, NMI and Stat5A expression is compromised that reduces the check on ISG20 expression. Increased ISG20 promotes invasive progression that leads to metastatic colonization at a secondary site.

Our results indicate that STAT5A, together with NMI protein expression in primary breast cancer tumors, is reduced compared to adjacent normal breast tissue. Also, that these proteins showed much reduced expression in metastatic tissues compared to their matched primary tumors. This is suggestive of negative influence of STAT5A-NMI axis on tumor progression (Fig. 7). Our investigation of STAT5A super enhancers related mammary-specific genes^17^ in the context of NMI modifications has revealed a group of genes that are potential downstream effectors of this signaling axis. We found the expression of ISG20 to be inversely correlated with NMI expression in NMI knockout mammary tumor mouse model and as well in human breast cancer cell lines. ISG20 (a.k.a. HEM45: HeLa estrogen-modulated, band 45) is an RNA exonuclease that has been studied to have a broad antiviral activity. Expression of ISG20 can be regulated by both type I and II interferons (IFNs) and it plays a role in mediating interferon’s antiviral activities^25,45,46^. Pentecost group, showed that ISG20 mediates estrogen controlled cellular proliferation and differentiation^47^. ISG20 was also found to support tumor progression in hepatocellular carcinoma, glioma, oral cancer, and renal cell carcinoma^48–51^. However, the details of regulation and functional role of ISG20 in cancers, specifically in breast cancer still remain unknown. We found that miR-17/92 cluster, specifically has-miR-20a, downstream of STAT5A controls ISG20. We observed that ISG20 expression is significantly elevated in metastatic lymph nodes tissues compared to its matched primary tumors; this suggests that ISG20 might have a role in the complex process of breast cancer metastasis. Our findings revealed that increased ISG20 promotes invasive phenotype of human breast cancer cells. At this point it is difficult to speculate if ISG20 has any specific role in the process of mammary development. Based on our observations, we rationalize that NMI and STAT5A play a critical role in mammary development. In cancer, expression and functional loss of these proteins allows ISG20 expression, and that promotes the invasive phenotype.

## Supplementary information

Supplementary Figure Legends

SI-Materials and Methods

Supplementary Figure 1

Supplementary Figure 2

Supplementary Figure 3
