# Supplementary material for: Disruption of STAT5A and NMI signaling axis leads to ISG20-driven metastatic mammary tumors
Source: Oncogenesis. 2021 Jun 2;10(6):45. doi: 10.1038/s41389-021-00333-y (PMC8172570; doi:10.1038/s41389-021-00333-y)
Supplement: Supplementary file 1 — Supplementary Figure Legends [file 41389_2021_333_MOESM1_ESM.docx]

**Supplementary Figure legends**

**Supp. FIG. 1**

Immunofluorescence staining for NMI (Green) and STAT5A (red) in control and shNMI HC11 cells that was embedded in Matrigel without stimulation with DIP. DAPI was used to stain the nucleus. Pictures show loss of NMI and STAT5A overall signal in shNMI cells.

**Supp. FIG. 2**

**a.** Fold expression changes of STAT5A using RTq-PCR in MDA-MB-468 NT control and siSTAT5A. **b.** Fold expression changes of Hsa-miR-17-3p, Hsa-miR-17-5p, Hsa-miR-20a-3p and Hsa-miR-20a-5p using RTq-PCR in MDA-MB-468 NT control and siSTAT5A cells, data shows a significant reduction in the levels of Hsa-miR-20a-3p and Hsa-miR-20a-5p (p=0.004, 0.014) respectively. **c.** Fold expression changes of ISG20 using RTq-PCR in MDA-MB-468 NT control and siSTAT5A, showing a significant increase in ISG20 RNA expression (p=0.0005). **d.** Western blot analysis of ISG20 and STAT5A proteins in MDA-MB-468 control and siSTAT5A cells. **e.** Fold expression changes of miR17-92 cluster members using RTq-PCR in MDA-MB-468 vector control and miR17-92 overexpressed cells, data shows a significant increase in the levels of most of the cluster members. **f.** Western blot analysis of ISG20 protein in MDA-MB-468 control and miR17-92 overexpressed cells, ISG20 level is shown to be reduced after overexpressing the miR17-92 cluster.

**Supp. FIG. 3**

**a.** Representative ISG20 IHC staining in normal mammary tissues. Arrows point out the expression pattern of ISG20 in the mammary acini. **b.** Western blot analysis confirming ectopic overexpression of ISG20 in MDA-MB-468 and MDA-MB-231 cells. Compared to control and ISG20 protein expression was significantly higher in MDA-MB-231 and MDA-MB-468 lentivirus transduced cells. **c.** MDA-MB-231 control and ISG20 overexpressing cells were embedded in Matrigel for 5 days, arrows point to invasive 3D organoids outgrowth of MDA-MB-231 ISG20 cells. **d.** Measurement of area (µm^2^) and circularity of 3D organoids structures of MDA-MB-231 ISG20 cells. MDA-MB-231 ISG20 cells show a significantly lower circularity than controls (p=<0.0001), this indicates more invasive outgrowth of these cells. MDA-MB-231 cells showed a significant larger area compared to control (p=0.02). **e.** Western blot analysis of baseline ISG20 expression in MDA-MB-231 control cells and shISG20 cells. **f.** Representative images for control or shISG20 MDA-MB-231 cells Invading through Matrigel invasion chamber, graphs represent significantly lower invaded cells/field in MDA-MB-231 shISG20 compared to control (p=<0.002).
