# Supplementary material for: Disruption of STAT5A and NMI signaling axis leads to ISG20-driven metastatic mammary tumors
Source: Oncogenesis. 2021 Jun 2;10(6):45. doi: 10.1038/s41389-021-00333-y (PMC8172570; doi:10.1038/s41389-021-00333-y)
Supplement: Supplementary file 2 — SI-Materials and Methods [file 41389_2021_333_MOESM2_ESM.docx]

**Materials and methods**

**Cell culture**

HC11 cells were obtained from ATCC (CRL-3062) and maintained in RPMI 1640 (ThermoFisher) with 10% FBS (ThermoFisher), 20ng/ml EGF (Sigma) and 5μg/mL insulin (Sigma). T47D cells were maintained in RPMI 1640 with 10% FBS with 5μg/mL insulin. MDA-MB-231 and MDA-MB-468 cells were cultured in DMEM F12 media (ThermoFisher) supplemented with 5% FBS. Mycoplasma-free cell lines were used in all our experiments.

**HC11 differentiation**

To differentiate HC11 cells, EGF was removed from the media 24 hours prior to the addition of DIP [RPMI 1640 media containing 100nM dexamethasone (Tocris), 5μg/mL insulin, and 5μg/mL ovine prolactin (NIDDK-oPRL-21 was obtained from Dr. A.F. Parlow at the National Hormone and Pituitary Program, Harbor-UCLA Medical Center, Torrance, CA)]. DIP media was changed every 24 hours. Differentiation process is completed by 72 hours (14).

**Immunofluorescence staining**

Cells were fixed using 4% PFA for 20 min at room temperature (RT), washed 3 times with PBS, then permeabilized using 0.1% Triton in PBS for 10 min at RT. Cells were then incubated in primary antibody for 3h at RT, followed by secondary antibody for 1h at RT, then mounted with DAPI (Vector Laboratories). Images were captured using a Nikon A1R HD Confocal Microscope (Nikon) using 40X lens and analyzed using NIS-Elements AR 5.20.02 software.

**Antibodies**

Antibody for E-Cadherin (Cell signaling) was used at 1:200 dilution. Mouse Polyclonal NMI antibody PA8076 (aa-63:80, described previously (8)) was used for western blot at 1:1000 and for IF at 1:100 dilution. PA7977 (aa-13:28, described previously(8)) was used for IHC at 1:200 dilution. Human NMI (Millipore Sigma) was used at 1:5000 dilution for WB and 1:3000 for IHC. STAT5A antibody (Thermofisher) was used at 1:1000 dilution for WB, 1:250 for IF and 1:500 for IHC. Phospho-STAT5 (Tyr694) antibody (Cell signaling) was used at 1:1000 dilution. ISG20 antibody (ABCAM) was used at 1:1000 dilution for WB and 1:3500 for IHC.

**Mammary differentiation in 3D culture**

Growth factor reduced Matrigel (70µL, Corning) was added to wells of 8 chambered slides (Millicell EZ slide Millipore) and incubated for 45 min at 37°C to solidify. Cells (5000) were added to each well in 100 µL regular media for the undifferentiated group and EGF-free media for the differentiation group. After 90 min, 100 µL of assay media (10% Matrigel) was used for overlay. The spheroids were allowed to grow for 48 hr and then 200 µL of either DIP or complete assay media was added (containing 5% Matrigel) to each well. Media were changed once every 2 days and the spheroids were allowed to grow for 4-5 days.

**Transfection**

NMI was silenced using SMARTvector mCMV-TurboRFO shRNA plasmid (Dharmacon). Cells were transfected using Lipofectamine 2000 (Invitrogen) according to manufacturers’ protocol and selected with puromycin (1µg/mL). miR17-92 expression plasmid (pcDNA3.1/V5-His-TOPO-mir17-92) was a gift from Joshua Mendell (Addgene 21109) (15)

ISG20 was silenced using GIPZ shRNA system (Dharmacon). Cells were transfected using Lipofectamine 2000 (Invitrogen) according to manufacturers’ protocol and selected with puromycin (0.5 µg/mL).

**Differentiation trajectory expression**

The data of [“Differentiation dynamics of the developing mammary gland revealed by single-cell RNA-sequencing”](https://www.nature.com/articles/s41467-017-02001-5) were interactively browsed for genes of interest using publicly available database at <https://marionilab.cruk.cam.ac.uk/mammaryGland/>.(16)

I**mmunostaining**

Immunohistochemical detection was performed using the Dako Envision Dual Link System-HRP system according to manufacturer’s protocol. Sections from murine tissues (5 µm) were immunostained with the NMI or STAT5A or ISG20 antibodies at previously stated concentrations. Citrate buffer antigen retrieval was used for ISG20. Cells or tissue sections were then incubated in primary antibody overnight at 4°C followed by secondary antibody for 1h at RT then covered using mounting medium fluorescence with DAPI (Vector lab). Images were captured using a Nikon Eclipse Ti inverted microscope (Nikon) using 40X lens and analyzed using NIS-Elements AR 5.20.02 software. Staining was assessed under light microscopy using immunoreactive scoring (IRS). Nuclear staining intensity was assessed using ImageJ Plug in IHC profiler (17, 18).

**Luciferase reporter assay**

Cells (20,000 cells per well) were plated at in a 96-well plate (Corning) and then transfected with 200 ng of β-Casein or STAT5A-RE reporter plasmids using Lipofetamine 2000 (Invitrogen). Twenty-four hours after transfection, media was changed and Prolactin (500ng/ml) or control was added to the STAT5A-RE group. The experiment was terminated after 30 min for measuring luciferase activity using the Luciferase Assay System (Promega) and a GloMax 20/20 Luminometer (Promega) according to manufacturer's protocol. Experiments were performed in triplicate and normalized to total protein content as measured by Precision Red assay (Cytoskeleton). β-Casein reporter has been previously characterized by Charles Clevenger’s group (19). pGL4 STAT5 RE luciferase reporter vector was obtained from Promega.

**MicroRNA** **array**

Exiqon miRCURY LNA microRNA array; 7^th^ generation cat# 208500, batch 35001 was used for miRNA expression profiling (Qiagen). The samples were labeled using the miRCURY LNA™ microRNA Hi-Power Labeling Kit, Hy3™/Hy5™ and hybridized on the miRCURY LNA™ microRNA Array. Following normalization of the quantified signals (background corrected) using the global Lowess regression algorithm, an unsupervised as well as supervised data analysis was performed.

**Transfection of miRNA mimics**

Cells (200,000/well) were cultured overnight in 6 well plate. Transfection was done using Lipofetamine 2000 reagent (Life Technologies) and mirVana miRNA Mimics (hsa-miR-20a-5p, hsa-miR-20a-3p, hsa-miR-17-5p, hsa-miR-17-3p, Life Technologies) at 100nM. Fresh media was added after 24 hours of transfection, and protein was collected for analysis 48 hours after transfection. (), mirVana miRNA

**Lentiviral transduction**

EX-A8597-Lv217 (for ISG20 overexpression) and empty vector pReceiver-Lv217 were purchased from Genecopoeia. Recombinant lentivirus was produced using Lenti-Pac HIV expression packing kit (Genecopoeia) according to manufacturer’s protocol. 2x10^5^ cells were cultured in 6 well plate overnight, 0.5 ml of virus suspension diluted in complete media with polybrene at final concentration of 8µg/ml.

**Invasion assay**

BD Biocoat Matrigel invasion chambers (Corning, 8.0µm pore size) were allowed to warm at room temperature. Warm, serum-free growth medium (500µl) was added into the inserts and the inserts were allowed to rehydrate (2 hr, 37⁰C, 5% CO_2_). Media was carefully removed and then 15,000 cells (in 500µl serum-free medium) were added to each insert. Growth medium (750µl, serum-free) containing 10µg/ml fibronectin was added to the lower wells. Cells were incubated in a 37⁰C / 5% CO_2_ incubator for 12 hr. and thereafter fixed in 4% paraformaldehyde (10 min) and stained with 0.5% crystal violet (Difco Laboratories) for 15 min. Filters were rinsed in water and air dried. Experiment was done in triplicate and 4 images per chamber were captured using Nikon Eclipse E200LED microscope using 20X lens.

**Migration assay**

Cell culture insert filters (8 µM pore-sized, BD Falcon) were coated with 600µl of 6ng/ml of gelatin overnight then rehydrated with 150µl warm serum-free media RT for 90 min. Media was carefully removed, and the inserts were placed in a well with 750µl of serum free media. Cells (50,000) in 500µl serum free media were seeded in each chamber and incubated for 4 hr (37⁰C / 5% CO_2_). Cells were fixed in 4% paraformaldehyde for 10 minutes and stained with 0.5% crystal violet for 15 minutes. Filters were rinsed in water and air dried. Experiment was done in triplicate and 4 images per insert were captured using Nikon Eclipse E200LED microscope using 20X lens.

**RNA Isolation and Real Time PCR**

Total RNA was isolated from cultured cells using RNeasy Mini Kit (Qiagen). RNA was quantitated and assessed using spectrophotometry (NanoDrop Lite, Thermo Scientific). Total RNA (1 μg) was transcribed into complementary DNA using the High-Capacity cDNA Reverse Transcription kit (Applied Biosystems). Quantitative real-time PCR was performed with TaqMan Fast Advanced Master Mix (Applied Biosystems) in a Step-one Plus (ThermoFisher) real-time PCR detection system.

For miRNA detection, RNA was harvested with TRIzol reagent and precipitated with isopropanol overnight. Total RNA (1 μg) was transcribed into complementary DNA using miSCRIPT II RT kit (Qiagen). Quantitative real time PCR was performed with QuantiTect SYBR Green PCR Kit (Qiagen) using miScript primer assay (Qiagen).

**TCGA Breast cancer patient data analysis**

RNA sequencing data (IlluminaHiSeq) of 1247 breast cancer primary tumors from TCGA Breast Cancer was accessed from public data portal (https://xenabrowser.net) in August 2020. Data were extracted for analysis and ISG20 RNA gene expression was compared between normal tissue and breast tumor tissue. T-test was used for statistical analysis using GraphPad Prism (GraphPad Software, La Jolla, CA). 10- Year KM overall survival and progression free interval analysis were extracted for analysis from the same data set and stratified by median into high and low STAT5A expression. Comparisons were considered statistically significant for p-value < 0.05.

**Pulmonary Metastasis Assay (PuMA)**

MDA-MB-231 control and ISG20 cells (1x10^5^) cells were injected into the tail vein of nude mice and allowed to circulate for a period of 15 minutes after which the mice were humanely euthanized. The lungs were then inflated with a low melt agarose media mixture and allowed to cool down to allow agarose to solidify. Lungs were then excised from the mouse for sectioning with a scalpel. 12-16 sections were cultured on Surgifoam for a period of approximately 31 days, flipping the lung sections every other day and changing the media every three to four days as needed. Assay procedure was adopted from previous a report by Mendoza et al. (20). Fluorescent images were taken of each lung section every 3 days to evaluate metastatic burden using Eclipse TE2000-U microscope (Nikon). Representative images for GFP positive foci were captured at 4X magnification. Images were analyzed using ImageJ-FIJI software and area corrected total cell fluorescence (CTCF) was calculated according. CTCF = Integrated Density – (Area of selected cell X Mean fluorescence of background readings).

**Graphical scheme**

Graphical scheme was created using Servier Medical Art templates (https://smart.serv ier.com.) and Microsoft power point.
