# Supplementary figures and images for: Disruption of STAT5A and NMI signaling axis leads to ISG20-driven metastatic mammary tumors
Source: Oncogenesis. 2021 Jun 2;10(6):45. doi: 10.1038/s41389-021-00333-y (PMC8172570; doi:10.1038/s41389-021-00333-y)

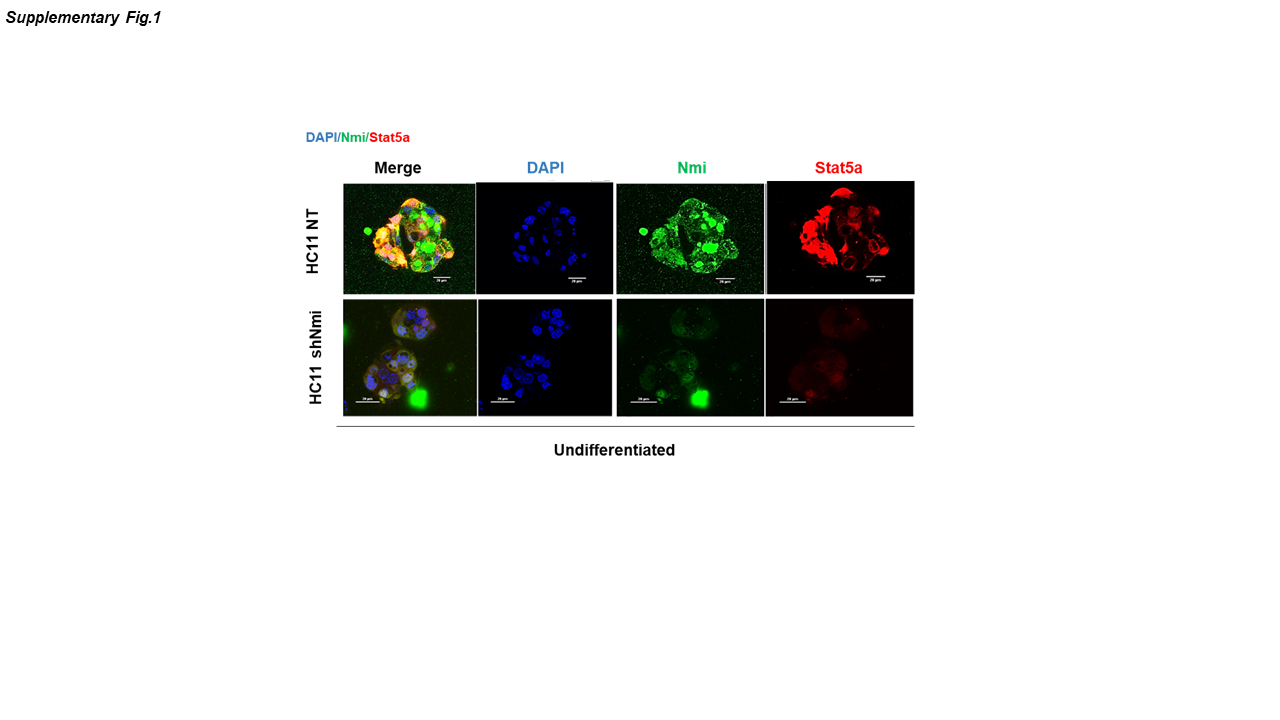

Supplement: Supplementary file 3 — Supplementary Figure 1 [file 41389_2021_333_MOESM3_ESM.tif]

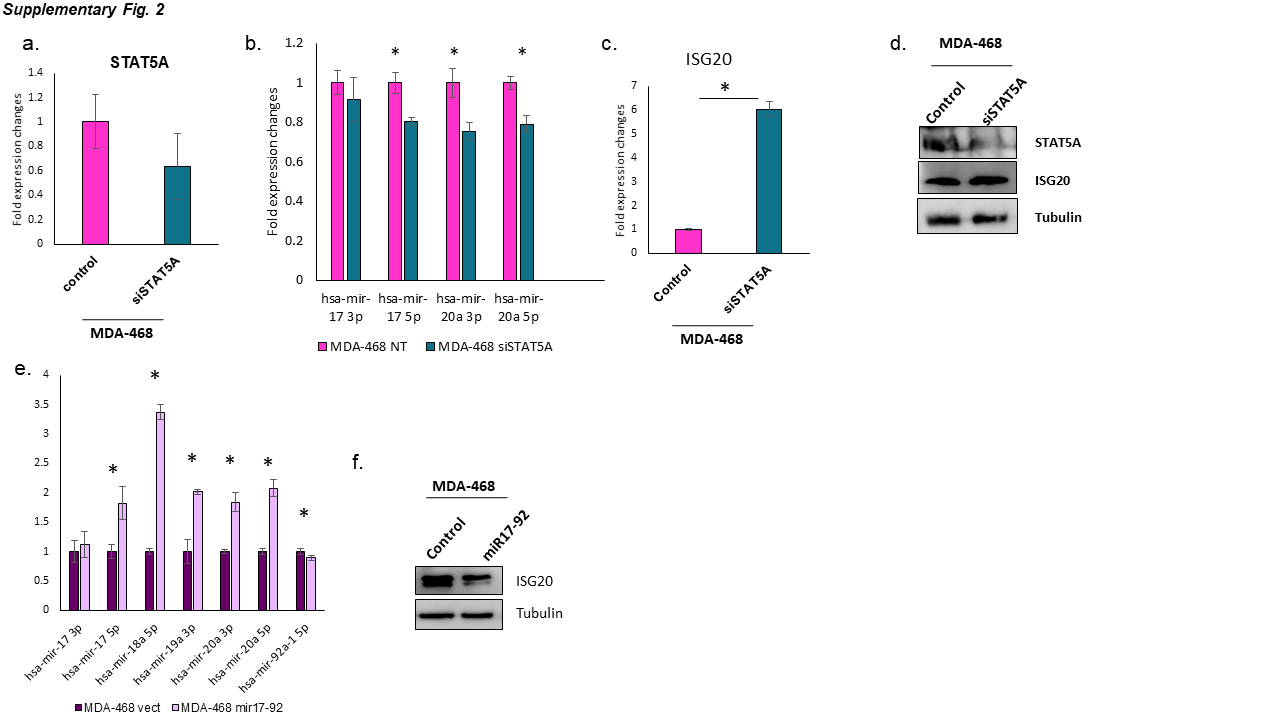

Supplement: Supplementary file 4 — Supplementary Figure 2 [file 41389_2021_333_MOESM4_ESM.tif]

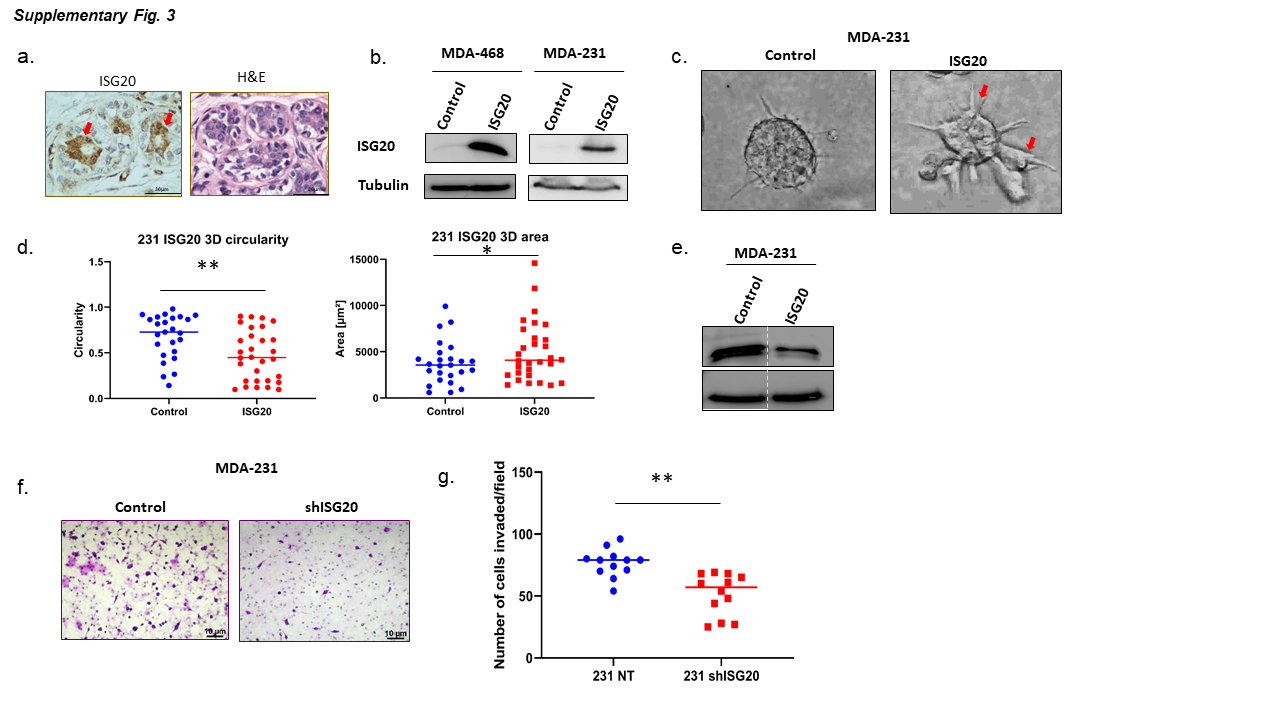

Supplement: Supplementary file 5 — Supplementary Figure 3 [file 41389_2021_333_MOESM5_ESM.tif]
